# Immune response in COVID-19: addressing a pharmacological challenge by targeting pathways triggered by SARS-CoV-2

**DOI:** 10.1038/s41392-020-0191-1

**Published:** 2020-05-29

**Authors:** Michele Catanzaro, Francesca Fagiani, Marco Racchi, Emanuela Corsini, Stefano Govoni, Cristina Lanni

**Affiliations:** 1 Department of Drug Sciences (Pharmacology Section), University of Pavia, V.le Taramelli 14, 27100 Pavia, Italy; 2 Scuola Universitaria Superiore IUSS Pavia, P.zza Vittoria, 15, 27100 Pavia, Italy; 3 Laboratory of Toxicology, Department of Environmental and Political Sciences, Università Degli Studi di Milano, Via Balzaretti 9, 20133 Milano, Italy

**Keywords:** Inflammation, Infectious diseases

## Abstract

To date, no vaccines or effective drugs have been approved to prevent or treat COVID-19 and the current standard care relies on supportive treatments. Therefore, based on the fast and global spread of the virus, urgent investigations are warranted in order to develop preventive and therapeutic drugs. In this regard, treatments addressing the immunopathology of SARS-CoV-2 infection have become a major focus. Notably, while a rapid and well-coordinated immune response represents the first line of defense against viral infection, excessive inflammatory innate response and impaired adaptive host immune defense may lead to tissue damage both at the site of virus entry and at systemic level. Several studies highlight relevant changes occurring both in innate and adaptive immune system in COVID-19 patients. In particular, the massive cytokine and chemokine release, the so-called “cytokine storm”, clearly reflects a widespread uncontrolled dysregulation of the host immune defense. Although the prospective of counteracting cytokine storm is compelling, a major limitation relies on the limited understanding of the immune signaling pathways triggered by SARS-CoV-2 infection. The identification of signaling pathways altered during viral infections may help to unravel the most relevant molecular cascades implicated in biological processes mediating viral infections and to unveil key molecular players that may be targeted. Thus, given the key role of the immune system in COVID-19, a deeper understanding of the mechanism behind the immune dysregulation might give us clues for the clinical management of the severe cases and for preventing the transition from mild to severe stages.

## Introduction

The outbreak of the novel coronavirus disease 2019 (COVID-19), induced by severe acute respiratory syndrome coronavirus 2 (SARS-CoV-2), firstly found in Wuhan, in the Hubei province of China, in December 2019, has rapidly spread worldwide, becoming a global public health emergency. On 11th March 2020, the World Health Organization (WHO) declared COVID-19 a pandemic. As of 28 April 2020, WHO reports more than 2,8 million confirmed cases and 198 842 deaths worldwide (WHO, 2020, https://covid19.who.int). After the isolation of SARS-CoV-2, the viral genome was sequenced, thus facilitating diagnostic testing, epidemiologic tracking, as well as investigations on potential preventive and therapeutic strategies in the management of COVID-19. To date, despite the intense scientific effort demonstrated by more than 600 clinical trials currently underway (typing SARS-CoV-2 on clinicaltrials.gov), no vaccines or effective drugs have been approved to prevent or treat COVID-19 and the current standard care is supportive treatment. Therefore, based on the fast and global spread of the virus, urgent investigations are warranted in order to develop effective therapies. Within this context, treatments addressing the immunopathology of the infection have become a major focus.

## Virology and host-pathogen interaction

The new human-infecting SARS-CoV-2 is a positive-sense single-stranded RNA-enveloped virus belonging to CoV family.^1^ Among the six CoVs pathogenic to humans, four of them have been associated with mild respiratory symptoms,^2^ while two of them, SARS-CoV and the Middle East respiratory syndrome (MERS) CoV (MERS-CoV), whose epidemic outbreaks took place in 2002 and 2012 respectively, caused severe respiratory diseases in affected individuals.^2^ SARS-CoV-2 is the seventh identified CoV and, after SARS-CoV and MERS-CoV, the third zoonotic virus of CoVs that has been transmitted from animals to humans via an intermediate mammalian host.^3,4^ In particular, based on genetic analysis, Chinese horseshoe bats have been proposed to serve as natural reservoir hosts for SARS-CoV-2, similar to SARS-CoV and MERS-CoV.^4–6^ Moreover, genomic analysis indicates that SARS-CoV-2 is in the same beta-CoV clade as SARS-CoV and MERS-CoV.^1^ In particular, SARS-CoV-2 has been observed to share almost 80% of the genome with SARS-CoV^1,6,7^ and almost all encoded proteins of SARS-CoV-2 are homologous to SARS-CoV proteins.^1^ In contrast, SARS-CoV-2 has been found to be more distant from MERS-CoV, with only 50% identity.^1^ Moreover, the entry of SARS-CoV-2 into human host cells has been found to rely on the same receptor as SARS-CoV: the surface angiotensin-converting enzyme 2 (ACE2), which is expressed in the type II surfactant-secreting alveolar cells of the lungs.^8,9^ Consistently, despite amino acid variations at specific key residues, homology modeling revealed a structural similarity between the receptor-binding domains of SARS-CoV and SARS-CoV-2.^1^ However, further studies are necessary to compare SARS-CoV and SARS-CoV-2 affinities to ACE2 receptor that might explain the increased transmissibility and greater virulence of SARS-CoV-2 compared with SARS-CoV.^8^

Two independent groups provided key insights into the first step of SARS-CoV2 infection, by demonstrating that ACE2 host receptor is required for host cell entry of SARS-CoV-2.^8,10^ Noteworthy, the expression of ACE2 receptors is not only restricted to the lung, and extrapulmonary spread of SARS-CoV in ACE-expressing tissues has been demonstrated.^11–13^ Hence the same pattern may be expected for SARS-CoV-2, with most of human tissues, such as oral mucosa and gastrointestinal tract, kidney, heart, blood vessels expressing ACE2 receptors, particularly prone to SARS-CoV-2 infection.^14,15^ The viral entry of SARS-CoV-2 has been further found to be prevented by a clinically proven inhibitor of the cellular host type 2 transmembrane serine protease TMPRSS2 (camostat mesylate).^8^ Priming of the envelope-located trimeric spike (S) protein by host proteases, which cleave at the S1/S2 and the S2’ sites, has been described as a fundamental step for viral entry, and the host protease TMPRSS2 emerged as a key cellular factor necessary for the priming of S protein and for the consequent membrane fusion and viral internalization by endocytosis in the pulmonary epithelium.^8^ Hence, TMPRSS2 has been proposed as a potential target for clinical intervention^6,8^ and its inhibitor camostat mesylate, approved for human use in Japan to treat pancreatic inflammation, has attracted the attention of the scientific community. Currently, a randomized, placebo-controlled, phase IIa trial is investigating the use of camostat mesylate (NCT04321096) and is expected to run until December 2020, whereas another independent trial will start in June 2020 to evaluate the efficacy of camostat mesilate in combination with hydroxychloroquine in hospitalized patients with moderate COVID-19 infection (NCT04338906).

A detailed analysis of additional mechanisms of cellular viral infection for SARS-CoV-2 is still missing and would be fundamental to identify further potential biological substrates to target.

## Immunopathology of COVID-19

The majority of COVID-19 cases (about 80%) is asymptomatic or exhibits mild to moderate symptoms, but approximately the 15% progresses to severe pneumonia and about 5% eventually develops acute respiratory distress syndrome (ARDS), septic shock and/or multiple organ failure.^16,17^ As for SARS and MERS, the most common symptoms of COVID-19 are fever, fatigue, and respiratory symptoms, including cough, sore throat and shortness of breath.^16,18^

Notably, SARS-CoV-2 infection activates innate and adaptive immune response, thus sustaining the resolution of COVID-19. While a rapid and well-coordinated immune response represents the first line of defense against viral infection, excessive inflammatory innate response and dysregulated adaptive host immune defense may cause harmful tissue damage at both at the site of virus entry and at systemic level. The excessive pro-inflammatory host response has been hypothesized to induce an immune pathology resulting in the rapid course of acute lung injury (ALI) and ARDS occurring in SARS-CoV-2 infected patients.^16–18^ For example, the massive cytokine and chemokine release, the so-called “cytokine storm”, clearly reflects a widespread uncontrolled dysregulation of host immune defense. Thus, given the key role of the immune system in COVID-19, a deeper understanding of the mechanism behind the immune dysregulation, as well as of SARS-CoV-2 immune-escape mechanisms might give us clues for the clinical management of the severe cases and for preventing the transition from mild to severe stages. Moreover, although no within the goal of the present review, future investigations concerning the systemic effects of uncontrolled immune system on other physiological systems, such as the gastrointestinal tract, neuroendocrine, renal and cardiovascular are urgent.

### Immune response to SARS-CoV-2

Several studies highlight relevant changes occurring both in innate and adaptive immune system in COVID-19 patients. In particular, lymphocytopenia and a modulation in total neutrophils are common hallmarks and seem to be directly correlated with disease severity and death.^6,18^ In patients with severe COVID-19, a marked decrease in the levels of absolute number of circulating CD4^+^ cells, CD8^+^ cells, B cells and natural killers (NK) cells,^16,17,19^ as well as a decrease in monocytes, eosinophils and basophils has been reported.^19–21^ In addition, most of patients with severe COVID-19 displayed significantly increased serum levels of pro-inflammatory cytokines (e.g. IL-6, IL-1β, IL-2, IL-8, IL-17, G-CSF, GM-CSF, IP-10, MCP-1, CCL3, and TNFα).^20,22^ Although no direct evidence for pro-inflammatory cytokines and chemokines involvement in lung pathology in COVID-19 has been reported, an increase in serum cytokine and chemokine levels, as well as in neutrophil-lymphocyte-ratio (NLR) in SARS-CoV-2 infected patients has been correlated with the severity of the disease and adverse outcomes, suggesting a possible role for hyper-inflammatory responses in COVID-19 pathogenesis.^20^ Moreover, a recent multicenter retrospective cohort study analyzing data from the Early Risk Stratification of Novel Coronavirus Pneumonia (ERS-COVID-19) study (ChiCTR2000030494) showed that patients with COVID-19 had elevated high-sensitivity C-reactive protein (Hs-CRP) and procalcitonin serum levels, two major inflammation markers associated with high risks of mortality and organ injury.^23^

Noteworthy, MERS-CoV has been demonstrated to infect THP-1 cells, human peripheral blood monocyte-derived macrophages and dendritic cells, and SARS-CoV to directly infect macrophages and T cells,^24^ thereby inducing delayed but elevated levels of pro-inflammatory cytokines and chemokines.^25,26^ However, ACE2 receptor is only minimally expressed in monocytes, macrophages, and T cells in the lung, hence, the mechanism by which SARS-CoV directly infects immune cells is still unknown.^27^ Taking into account the similarities between SARS-CoV and SARS-CoV-2, it is likely that also this latter may infect monocytes and macrophages by a mechanism that has to be still unveiled. In this regard, it is possible that the virus may be capable to bind other specific receptors and/or other mechanisms of viral entry mode can be exploited by the virus.

As far as concerns the adaptive immunity, the novel SARS-CoV-2 has been demonstrated to mainly affect lymphocyte counting and balance. In particular, Li et al. reported that, compared with survivors, dead COVID-19 patients showed lower percentage and count in CD3^+^, CD4^+^, and CD8^+^ lymphocytes populations, strong predictive values for in-hospital mortality, organ injury, and severe pneumonia.^23^

In a retrospective, single-center study enrolling a cohort of 452 patients with COVID-19 in Wuhan, patients with severe COVID-19 displayed a significantly lower number of total T cells, both helper T cells and suppressor T cells.^20^ In particular, among helper T cells, a decrease in regulatory T cells, with a more pronounced reduction according to the severity of the cases, and in memory T cells has been observed, whereas the percentage of naïve T cells was found increased.^20^ Notably, naïve and memory T cells are essential immune components, whose balance is crucial for maintaining a highly efficient defensive response. Naïve T cells enable the defenses against new and previously unrecognized infection by a massive and tightly coordinated release of cytokines, whereas memory T cells mediate antigen-specific immune response. A dysregulation in their balance, favoring naïve T cells activity compared with regulatory T cells, could highly contribute to hyperinflammation. A reduction in memory T cells on the other hand could be implicated in COVID-19 relapse, since a number of recurrences has been reported in recovered cases of COVID-19.^6,28^ These data are consistent with results reported by Tan et al.^29^ Overall, the lymphopenia observed in COVID-19 patients may depend on the fact that SARS-CoV-2 may directly infect lymphocytes minimally expressing ACE2, leading to lymphocyte death or, alternatively, may directly damage lymphatic organs since they express ACE2 receptors.^29^ However, to date no data are available on lymph nodes and spleen shrinking and lymphocytes functionalities, hence such speculations need to be further investigated to confirm these hypotheses.

As far as concerns B cells, by using single-cell RNA sequencing to characterize the transcriptome landscape of blood immune cell subsets during the recovery stage of COVID-19, Wen et al.^30^ found significant changes in B cells. In particular, while the naïve B cells have been reported to be decreased, the plasma cells have been found remarkably increased in peripheral blood mononuclear cells.^30^ Moreover, several new B cell-receptor changes have been identified (e.g. IGHV3–23 and IGHV3–7).^30^ In addition, isotypes, including IGHV3–15, IGHV3–30, and IGKV3–11, previously used for virus vaccine development have been confirmed.^30^ The strongest pairing frequencies, IGHV3–23-IGHJ4, has been suggested to indicate a monoclonal state associated with SARS-CoV-2 specificity.^30^ Moreover, given the pivotal role of B cells in the control of infections, tracking the antibody seroconversion response is an important process for the clinical evaluation of infections. In COVID-19 patients, while serum samples from patients with COVID-19 showed no cross-binding to the S1 subunit of the SARS-CoV spike antigen, some cross-reactivity of serum samples has been observed from patients with COVID-19 to nucleocapsid antigens of SARS-CoV.^31^ Interestingly, this study reports that 96.8% of tested patients achieved seroconversion of IgG or IgM within 20 days after symptom onset with a titer plateaued within 6 days after seroconversion.^31^ Moreover, 100% of patients had positive virus-specific IgG approximately 17–19 days after symptom onset.^31^ Instead, 94.1% patients showed positive virus-specific IgM approximately 20–22 days after symptom onset.^31^

In addition to these observations about immunity, a critical aspect has to be raised concerning the ability to escape from anti-viral host defenses. Viral evasion of host immune response is in fact believed to play a major role in disease severity.^32^ As an example, SARS-CoV and MERS-CoV escape and suppress the signaling pathways mediated by type I Interferon (IFN), a key cytokine secreted by virus-infected cells to enroll nearby cells to heighten their anti-viral immune defenses.^33^ Based on genomic sequence comparison and on partial identity of SARS-CoV-2 with SARS-CoV, it is speculative that SARS-CoV-2 can adopt similar strategies to modulate the host innate immune response, thus evading immune detection and dampening human defenses.

### Inflammatory cytokine storm and lung damage

Mounting clinical evidence from severe COVID-19 patients suggests that extensive changes in the serum levels of several cytokines play a pivotal role in the pathogenesis of COVID-19.^22,34,35^ Such hypercytokinemia, the so-called “cytokine storm”, has been proposed as one of the key leading factors that trigger the pathological processes leading to plasma leakage, vascular permeability, and disseminated vascular coagulation, observed in COVID-19 patients, and accounting for life-threatening respiratory symptoms.^17^ Huang et al.^16^ found that plasma concentrations of IL-1β, IL-1ra, IL-7, IL-8, IL-9, IL-10, basic FGF, G-CSF, GM-CSF, IFN-γ, IP-10, MCP-1, MIP-1α, MIP-1β, PDGF, TNFα, and VEGF were higher in both ICU (intensive care unit) patients and non-ICU patients than in healthy adults. Moreover, when comparing ICU and non-ICU patients, plasma concentrations of IL-2, IL-7, IL-10, G-CSF, IP-10, MCP-1, MIP-1α, and TNFα were higher in ICU patients than non-ICU patients, thus indicating that the cytokine storm might be correlated with disease severity.^16^ Another study on a small set of patients with severe COVID-19 pneumonia, found 15 cytokines (IFN-α2, IFN-γ, IL-1ra, IL-2, 4, 7, 10, 12 and 17, chemokine IP-10, as well as G-CSF and M-CSF) associated with lung injury based on Murray score.^35^ Evidence from literature indicates that the cytokine storm observed in COVID-19 resembles that occurring in Cytokines Release Syndrome (CRS), a form of systemic inflammatory response syndrome, and in secondary haemophagocytic lymphohistiocytosis (sHLH), an hyperinflammatory syndrome characterized by fulminant and fatal hypercytokinemia with multiorgan failure, mainly induced by viral infections.^22,36^ Therefore, as detailed below, existing pharmaceutical modulators of cytokines might be repurposed as therapeutic strategy to attenuate the hypercytokinemia in COVID-19 patients.

Interestingly, Gou et al.^37^ recently reported that the disruption of gut microbiome features by host and environmental factors may predispose healthy individuals to abnormal inflammatory response observed in COVID-19. In particular, the authors constructed a blood proteomic risk score for the prediction of COVID-19 progression to clinically severe phase and observed that core gut microbiota features were significantly correlated with proinflammatory cytokines in a set of 366 individuals, using a machine learning model.^37^ Specifically, *Bacteroides* genus, *Streptococcus* genus and *Clostridiales* order have been negatively correlated with most of the tested inflammatory cytokines, whereas *Ruminococcus* genus, *Blautia* genus and *Lactobacillus* genus have been positively associated.^37^ Moreover, fecal metabolomics analysis indicated some potential amino acid-related pathways (e.g. aminoacyl-tRNA biosynthesis pathway, arginine biosynthesis pathway, and valine, leucine and isoleucine biosynthesis pathway) that correlate core microbial features with host inflammation among 987 participants.^37^ Thus, the core intestinal microbiological characteristics, along with its related metabolites, should be further investigated as potential predictors for the individual susceptibility to COVID-19 progression and severity and might represent potential targets for the prevention of susceptible populations, as well as for the development of therapeutic approaches to manage COVID-19.

## Putative signaling pathways triggered by SARS-CoV-2

**Fig. 1:**
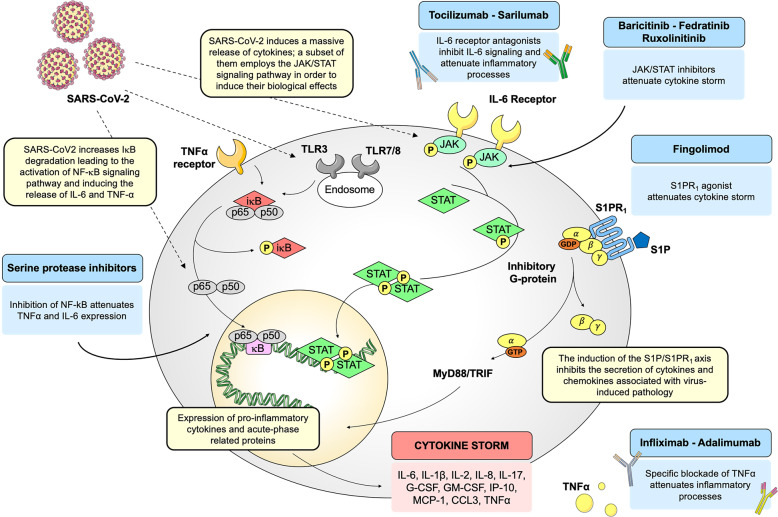
Schematic representation of SARS-CoV-2-driven signaling pathways and potential drug targets. Schematic representation of host intracellular signaling pathways induced by SARS-CoV-2 infection. Selected drugs, acting on these pathways, are repurposed to manage the cytokine storm induced by the viral infection. SARS-CoV-2, severe acute respiratory syndrome coronavirus 2; IκB, inhibitor of nuclear factor κB; NF-κB, p65-p50, nuclear factor κB; IL-6, interleukin 6; IL-1β, interleukin 1β; IL-2, interleukin 2; IL-8, interleukin 8; IL-17, interleukin 17; G-CSF, granulocyte-colony stimulating factor; GM-CSF, granulocyte macrophage-colony stimulating factor; IP-10, IFN-γ-induced protein 10; MCP-1, monocyte chemoattractant protein 1; CCL3, chemokine (C-C motif) ligand 3; TNFα, Tumor necrosis factor α; JAK, Janus kinase; STAT, signal transducer and activator of transcription; S1P, sphingosine-1-phosphate; S1PR_1_, sphingosine-1-phosphate receptor 1; MyD88, myeloid differentiation primary response gene 88; TRIF, TIR-domain-containing adapter-inducing IFN-β

It is well-established that, upon binding of the viral spike protein to the host cells by the entry receptor ACE2, the viral RNAs, as pathogen-associated molecular patterns (PAMPs), are detected by the pattern recognition receptors, which include the family of Toll-like receptors (TLRs). In particular, for RNA virus such as CoVs, viral genomic RNA or the intermediates during viral replication, including dsRNA, are recognized by either the endosomal RNA receptors, TLR3 and TLR7/8, and the cytosolic RNA sensor, retinoic acid-inducible gene (RIG-I)/MDA5.^38^ Consistently, such TLRs have been found to activate different signaling pathways in human CD14^+^ monocytes, correlating with differential type I IFN and cytokine secretion involved in CD4^+^ T cells polarization.^38^ As a result of virus recognition, downstream transduction pathways, crucial for proper antiviral response, such as IRF3 (IFN regulatory factor-3), nuclear factor κB (NF-κB), JAK (Janus kinase)/STAT (signal transducer and activator of transcription) signaling pathways, are activated.^39^ The identification of the most relevant intracellular signaling pathways involved in the modulation of host immune systems may give important hints on how to overcome the infectious disease driven by SARS-CoV-2. In particular, taking into account the structural similarities of SARS-CoV-2 as well as the analogies in the infection mechanisms with pathogenic SARS-CoV, it is tempting to speculate that the viral infection may induce the activation of shared intracellular pathways, in particular of those mainly involved in the innate immune response. However, to date, it has to be demonstrated whether such sequence similarities between SARS-CoV and SARS-CoV-2 can be directly translated into similar biological outcomes. Taking into account such limitation, the identification of signaling pathways altered during viral infections may help to unravel the most relevant molecular cascades implicated in biological processes mediating viral infections and to unveil key molecular players that may be targeted. The advantage of targeting intracellular molecules rather than viral proteins is that their effect is not likely to be negated by mutations in the virus genome. In fact, antiviral drugs inhibiting virus replication may select for mutational escape, thus rendering the therapy ineffective. Thus, the modulation of the host immune response shows the potential advantage of exerting less-selective pressure on viral populations.^40^ Repurposing of existing drugs targeting specific signal transducers will be discussed as potential treatment options for the management of COVID-19, as schematized in Fig. 1.

### The NF-κB/TNFα signaling pathway

The transcription factor NF-κB is a critical regulator of both innate and adaptive immunity.^41^ Under basal conditions, NF-κB is retained in the cytoplasm by the inhibitory proteins (IκBs). A variety of cellular stimuli, including pathogens, induce IκB phosphorylation, ubiquitination and degradation by the proteasome, thereby promoting NF-κB nuclear translocation.^41^ In the nucleus, NF-κB induces the transcription of a wide spectrum of genes encoding pro-inflammatory cytokines and chemokines, stress-response proteins, and anti-apoptotic proteins. NF-κB activity is essential for survival and activation, and for initiating and propagating optimal immune responses.^42^ By contrast, the constitutive activation of the NF-κB pathway is often associated with inflammatory diseases, such as rheumatoid arthritis and asthma. Notably, the exacerbation of NF-κB activation has been reported to be implicated in lung inflammatory immunopathology induced by respiratory viruses, including SARS-CoV.^43,44^ Moreover, Wang and collaborators demonstrated that, in murine macrophages cell line (RAW264.7), the exposure to recombinant SARS-CoV spike protein induced a massive protein release of IL-6 and TNFα in a time- and concentration-dependent manner in the supernatants and that such increase in IL-6 and TNFα secretion relies on the activation of NF-κB signaling pathway.^45^ In fact, SARS-CoV spike protein has been associated with an increase in IκBα degradation, an essential step required for the activation of NF-κB signaling pathway.^45^ Accordingly, transfection with dominant-negative NIK, which inhibits NF-κB activation, produced a strong reduction in spike protein IL-6 and TNFα release in RAW264.7 cells, thus demonstrating that NF-κB is required for the induction of IL-6 and TNFα by SARS-CoV spike protein.^45^ Such in vitro data were consistent with results obtained in vivo, where treatments with drugs inhibiting NF-κB activation (such as caffeic acid phenethyl ester (CAPE), Bay 11–7082, and parthenolide) reduced inflammation by suppressing the mRNA expression of TNFα, CXCL2, and MCP-1 in the lung of SARS-CoV-infected mice. Moreover, pharmacological inhibition of NF-κB protected against pulmonary pathology and enhanced mice survival after SARS-CoV infection.^43^

In line with these findings, Smits et al. demonstrated that SARS-CoV-infected aged macaques show in the lungs an increase in NF-κB nuclear translocation, as a result of NF-κB activation, and developed a stronger host response to virus infection compared with young adult macaques, with a significant increase in the expression of pro-inflammatory genes mainly regulated by NF-κB.^44^

Taken together these data suggest that NF-κB inhibition might be an effective strategy to counteract pathogenic SARS-CoV. However, targeting NF-κB is an approach strongly limited by intrinsic pathways complexity. Molecules blocking NF-κB lack for specificity and interfere with NF-κB physiological roles in cellular homeostasis, resulting in increased risk of undesired side effects, such as a broad suppression of innate immunity.^46^ Moreover, within the context of viral infection, a major limitation of targeting NF-κB signaling depends on the ability of viruses to efficiently escape, by encoding proteins specifically blocking this pathway.^46^ Thus, a promising strategy may rely on directly targeting the downstream effectors of the pathway, such as TNFα, whose expression is mainly controlled by NF-κB transcriptional activity. While TNFα is known to play a key role in the coordination and development of the inflammatory response, especially in the acute phase, long-lasting and excessive production of TNFα may become less effective by possibly altering TNF/TNF receptor signaling threshold which, after an initial wave of NF-κB activation, favors sustained basal NF-κB activity.^47^ In addition, despite many other pro-inflammatory cytokines and mediators are involved in the cytokine storm, specific blockade of TNFα has been reported to be clinically effective in several pathological conditions. Accordingly, TNFα blockers, such as infliximab and adalimumab, have been successfully used for the treatment of several immune-mediated disorders, such as psoriasis, rheumatoid arthritis, inflammatory bowel diseases and ankylosing spondylitis.^48,49^ Hence, anti-TNFα monoclonal antibodies are likely to attenuate inflammatory processes occurring in COVID-19, reducing the release of other inflammatory-exacerbating mediators. Indeed, when an anti-TNFα is administrated in patients with active rheumatoid arthritis, it has been demonstrated to induce a rapid decrease of a broad spectrum of cytokines (e.g. IL-6 and IL-1), as well as of others acute-phase related proteins and vascular permeability factor.^50–52^

Furthermore, the envelope viral spike protein of SARS-CoV has been found to promote the activity of TNFα-converting enzyme (TACE)-dependent shedding of ACE2 receptor, which is a fundamental step for virus entry into the cell.^53^ Thus, TNFα blockers represent effective therapeutic tools to counteract SARS-CoV infection by exerting a dual mechanism of action: attenuation of inflammation and inhibition of viral infection.^45^ However, warnings about the potential increased risk of bacterial and fungal superinfections due to anti-TNFα therapy have to be taken into account.^54^ Taking into account the sequence similarities between SARS-CoV and SARS-CoV-2 and the strong limitation in directly inhibiting NF-κB, to date, a clinical trial investigating adalimumab for the management of COVID-19 has been registered in the Chinese Clinical Trial Registry (ChiCTR2000030089) and is expected to run until August 2020. However, further investigations concerning the use and safety of TNFα-blockers in COVID-19 patients are urgently needed.

In addition, concerning a potential intervention on NF-κB signaling pathway, serine protease inhibitors of trypsin-like serine proteases (e.g. camostat mesylate, nafamostat mesylate, gabexate mesylate, ulinastatin), used for the treatment of pancreatitis, disseminated intravascular coagulation, and anticoagulant for hemodialysis,^55,56^ have been found to inhibit viral replication^57,58^ and to attenuate inflammatory processes in different pathological contexts, such as asthma, chronic allergic pulmonary inflammation, and inflammatory myocardial injury.^59–62^ For instance, nafamostat mesylate and gabexate mesylate have been demonstrated to attenuate allergen-induced airway inflammation and eosinophilia in mouse model of allergic asthma,^61^ thus reducing mast cell activation, eosinophils infiltrations in the lung, and *Dermatophagoides pteronyssinus*-driven IL-4 and TNFα production in bronchoalveolar lavage fluid.^61^ Furthermore, treatment with nafamostat mesylate downregulated the expression of IL-1β, TNFα, IL-6, eotaxin, inducible NO synthase (iNOS), CD86, and NF-κB activation, but enhanced the expression of IL-12 and IL-10 in *Dermatophagoides pteronyssinus*-driven IL-4 and TNFα production in bronchoalveolar lavage fluid.^61^ Moreover, gabexate mesylate has been found to inhibit LPS-induced TNFα production in human monocytes by blocking both NF-κB and mitogen-activated protein kinase activation.^63^ Thus, the pharmacological profile of serine protease inhibitors, as inhibitors of complement pathways and broad-spectrum anti-inflammatory agents, provide a strong rationale for their use in the management of COVID-19. However, the specific mechanism of action through which serine protease inhibitors induce their anti-inflammatory effects is still unknown.

### The IL-6/JAK/STAT signaling pathway

**Fig. 2:**
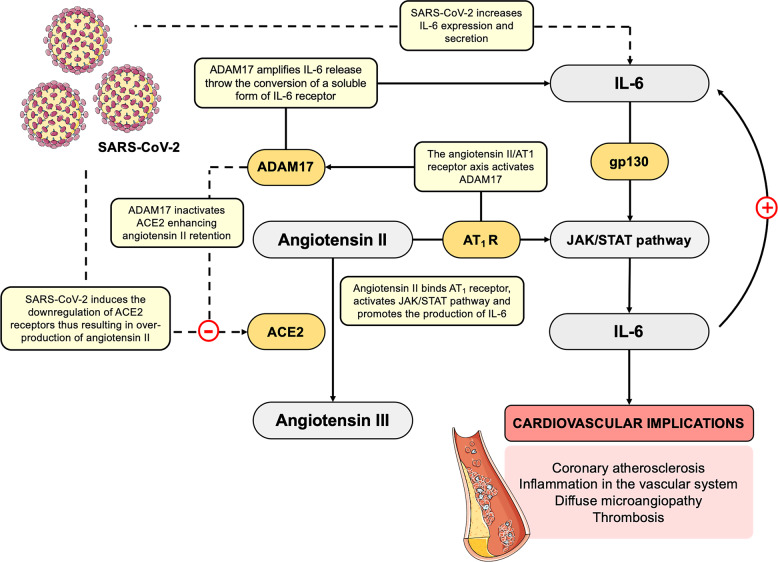
Hypothetical mechanism by SARS-CoV-2 in establishing an inflammatory feedback loop between IL-6 and angiotensin II. Cytokine IL-6 has been found increased in COVID-19 patients, thus suggesting a direct role of SARS-CoV-2 in a massive cytokine release. IL-6 is able to activate a soluble form of IL-6 receptor interacting with gp130, thereby promoting the downstream activation of JAK/STAT signaling, and thely production of IL-6. Moreover, SARS-CoV-2 has been directly related with the occurrence of cardiovascular implications, such as coronary atherosclerosis, inflammation in the vascular system and diffuse microangiopathy with thrombosis. Synthesis and secretion of IL-6 are directly implicated in cardiovascular damages. Indeed, IL-6 production is also induced by angiotensin II in AT_1_/JAK/STAT-dependent manner. As observed in SARS-CoV, also SARS-CoV-2 may be hypothesized to downregulate ACE2 expression, thus resulting in over-production of angiotensin II by the related enzyme ACE. In turn, increased angiotensin II enhances IL-6 production via JAK/STAT pathway, thus establishing a positive inflammatory feedback loop, ultimately resulting in the exacerbation of vascular and lung injuries. Moreover, the angiotensin II/AT_1_ receptor axis activates ADAM17 that cleavages and inactivates ACE2, enhancing angiotensin II retention. In addition, ADAM17 induction has been found to process the membrane form of IL-6Rα to the soluble form (sIL-6Rα), followed by the gp130-mediated activation of STAT3 via the sIL-6Rα-IL-6 complex in a variety of IL-6Rα-negative non-immune cells. The IL-6 amplifier promotes the production and secretion of several pro-inflammatory cytokines and chemokines, such as IL-6, sustaining the IL-6 amplifier-driven positive feedback. SARS-CoV-2, severe acute respiratory syndrome coronavirus 2; IL-6, interleukin 6; ACE2, angiotensin-converting enzyme 2; AT_1_, angiotensin II receptor type 1; JAK, Janus Kinase; STAT, Signal Transducer and Activator of Transcription; ADAM17, A Disintegrin And Metalloproteinase domain-containing protein 17

First discovered as the primary mediator of intracellular signaling induced by IFN in hematopoietic and immune cells, the JAK/STAT signaling is a key pathway transducing extracellular signals transmitted by a large number of cytokines, lymphokines and growth factors. In particular, a subset of cytokines employs the JAK/STAT signaling pathway in order to induce their biological effects. Notably, one of the major activators of JAK/STAT signaling is the cytokine IL-6, which has been reported to be dramatically increased in COVID-19 patients,^20,22^ with a strong implication in acute inflammation and cytokine storm. In particular, IL-6 has been reported to activate numerous cell types expressing the glycoprotein (gp130) receptor and the membrane-bound IL-6 receptor, as well as a soluble form of IL-6 receptor interacting with gp130, thereby promoting the downstream activation of JAK/STAT signaling.^64^ In turn, the activation of JAK/STAT pathway has been reported to stimulate the production of IL-6.^65^ Such signaling pathway has been reported to be aberrantly activated in patients with chronic inflammation conditions, such as arthritis rheumatoid, and it is likely that its excessive overactivation may also occur in COVID-19 patients, thereby exacerbating the host inflammatory response. Noteworthy, chronic elevation of circulating IL-6 has been widely recognized as a predictor for increased risk of cardiovascular events.^66,67^ Consistently, IL-6 is produced from several tissues, including activated macrophages and endothelial and smooth muscle cells, where it promotes the secretion of other cytokines and, among others, it stimulates MCP-1 secretion from macrophages to promote atherogenesis,^68^ increases the expression of cell adhesion molecules,^69,70^ and stimulates the proliferation and migration of vascular smooth muscle cells.^71^ Thus, the abnormal increase in IL-6 levels may be implicated, at least in part, in the occurrence cardiovascular diseases (e.g. coronary atherosclerosis, inflammation in the vascular system resulting in diffuse microangiopathy with thrombosis) observed in COVID-19 patients.^72^ Accordingly, the synthesis and secretion of IL-6 has been demonstrated to be induced by angiotensin II, which is locally produced by the inflamed vessels in a JAK/STAT-dependent manner.^73^ In particular, angiotensin II binding to Angiotensin II receptor type 1 (AT_1_ receptor) has been found to activate JAK/STAT pathway and to promote the downstream production of IL-6.^73,74^ Increased angiotensin II enhances IL-6 production in AT_1_/JAK/STAT-dependent manner, thus establishing a positive inflammatory feedback loop. Interestingly, the spike protein of SARS-CoV has been demonstrated to downregulate ACE2 expression, thus resulting in over-production of angiotensin II by the related enzyme ACE.^75,76^ In a similar way, it could be hypothesized that SARS-CoV-2 may downregulate ACE2 receptors, thus leading to an over-production of angiotensin II, in turn enhancing IL-6 production in AT_1_/JAK/STAT-dependent manner, and ultimately driving to vascular inflammation and lung injury, clinical signatures of COVID-19 (Fig. 2). Moreover, the angiotensin II/AT_1_ receptor axis has been reported to also activate both NF-κB and ADAM17.^77^ Notably, an important substrate for ADAM17 is ACE2, whose cleavage by ADAM17 has been reported to inactivate it, enhancing angiotensin II retention, thus leading to hypertension, cardiovascular remodeling, and other types of pathophysiology associated with enhancement of the renin-angiotensin system.^77^ Beside its implication in the shedding of ACE2 receptor, fundamental for virus entry,^53^ ADAM17 induction has been found to process the membrane form of IL-6 receptor α (IL-6Rα) to the soluble form (sIL-6Rα), followed by the gp130-mediated activation of STAT3 via the sIL-6Rα-IL-6 complex in a variety of IL-6Rα-negative non-immune cells.^78^ The activation of STAT3 has been reported to be required for the complete induction of NF-κB pathway.^78^ Thus, SARS-CoV-2 infection may activate both NF-κB and STAT3 signaling, which in turn can promote the IL-6 amplifier mechanism, required for the hyper-activation of NF-κB by STAT3, thereby inducing multiple inflammatory and autoimmune diseases.^78^ The IL-6 amplifier promotes the production and secretion of several pro-inflammatory cytokines and chemokines, such as IL-6, and the recruitment of lymphoid and myeloid cells, sustaining the IL-6 amplifier-driven positive feedback loop (as proposed by Hirano and Murakami)^79^ (Fig. 2). Furthermore, the metalloprotease ADAM17 has been found to mediate angiotensin II-induced EGFR (epidermal growth factor receptor) transactivation by generating the mature form of heparin-binding EGF-like growth factor in vascular smooth muscle cells, thus leading to vascular remodeling.^77^ Notably, EGFR transactivation is critical for angiotensin II-mediated cardiovascular complications.^77^ In this regard, the EGFR kinase inhibitor Erlotinib has been recently repurposed for the treatment of COVID-19, based on its capability to reduce the infectivity of a wide range of viruses.^80–82^ Beside its antiviral activity, the implication of EGFR transactivation in cardiovascular complications represent another theoretical foundation for the use of erlotinib in the management of COVID-19 patients.

Moreover, given the importance of angiotensin II/AT_1_ receptor axis, the attempt to use angiotensin II-receptor blockers as a therapeutic benefit in COVID-19 by targeting the host response to the virus has been made.^83^ However, their use needs to be deepened, since ACE inhibitors and angiotensin II-receptor blockers have been suggested to further increase the risk of COVID-19 infection by up-regulating ACE2.^84^ Whether patients affected by COVID-19 and hypertension, taking an ACE inhibitors or angiotensin II-receptor blockers, should switch to another antihypertensive drug is still a matter of debate, and further evidence is required.

Since IL-6 appears a key driver of cytokine storm and of its consequent detrimental effects, monoclonal antibodies against IL-6, such as tocilizumab and sarilumab, have been also proposed to dampen this process. Tocilizumab, a monoclonal antibody IL-6 receptor antagonist, approved for the treatment of rheumatoid arthritis and CRS, has been used in clinical practice in order to manage severe cases of COVID-19 and it has been included in the current Chinese national treatment guidelines (https://www.chinalawtranslate.com/wp-content/uploads/2020/03/Who-translation.pdf). To date, 40 clinical trials (typing COVID-19 and tocilizumab on clinicaltrials.gov and clinicaltrialsregister.eu) are underway to test tocilizumab, alone or in combination, in patients with COVID-19. Moreover, 18 clinical trials (typing COVID-19 and tocilizumab on clinicaltrials.gov and clinicaltrialsregister.eu) will study the efficacy and safety of another IL-6 receptor antagonist, sarilumab, approved for the treatment of rheumatoid arthritis in patients with COVID-19.

Beside monoclonal antibodies specifically targeting IL-6, approved drugs inhibiting IL-6/JAK/STAT signaling may represent a valuable tool. In particular, JAK signaling inhibitors, such as baricitinib, fedratinib, and ruxolitinib — approved for indications such as rheumatoid arthritis and myelofibrosis — have been reported to attenuate the host inflammatory response associated with massive pro-inflammatory cytokine and chemokine release.^85^ Based on this anti-inflammatory effect, they are likely to be effective against the consequences of the elevated levels of cytokines typically observed in patients with COVID-19.^80^ Among them, baricitinib, a selective inhibitor of JAK 1 and 2, has been predicted by crystallographic studies to inhibit two members of the numb-associated kinase family, such as AP2-associated protein kinase 1 (AAK1) and cyclin G-associated kinase (GAK), thus hindering viral endocytosis into lung cells, at the concentration approved for the treatment of arthritis rheumatoid.^86^ However, despite such undeniable advantages, the repurposing of baricitinib and, in general, of JAK inhibitors for the management of COVID-19 is debated. In particular, concerns arise mainly from evidence reporting that the activation of JAK/STAT pathway, mediated by IFNs, is required for the induction of many IFN-regulated genes, playing a pivotal role as innate early defense system against viral infections. The defensive role of JAK/STAT pathway is corroborated by evidence demonstrating that the majority of virus have developed escaping strategies, such as the production of viral-encoded factors blocking this pathway, which are recognized as crucial determinants of virulence.^87^ Therefore, inhibition of JAK/STAT signaling is likely to produce an impairment of IFN-related antiviral response, exacerbating SARS-CoV-2 infection. However, since several benefits, such as the blockage of virus entry and the attenuation of host excessive inflammatory response, as well as vascular and lung damage, provide a strong rationale for the use of baricitinib in the management of COVID-19 patients, the balance between positive and negative aspects of JAK/STAT signaling inhibition has to be still drawn up.

To date, several clinical trials are testing the efficacy and safety JAK inhibitors in COVID-19 patients (typing COVID-19 and JAK inhibitors on clinicaltrials.gov and clinicaltrialsregister.eu).

### The sphingosine-1-phosphate receptor 1 pathway

The sphingosine-1-phosphate (S1P) 1 has emerged as a crucial signaling lipid regulator of inflammation and immune response, including lymphocyte trafficking, vascular integrity, and cytokine and chemokine production.^88^ Beside S1P role of second messenger during inflammation, most of S1P effects on innate and adaptive immunity are mediated by its binding to five G-protein-coupled receptors (S1PRs_1–5_), which are differentially expressed in tissues.^88^ Among them, S1P_1_ receptor is ubiquitously expressed and coupled with a G inhibitory protein.^89^ The activation of S1P_1_ receptor is associated with Ras/ERK, PI3K/Akt/eNOS, and PLC/Ca^2+^ downstream pathways.^89^ Notably, under physiological and pathological conditions, the S1P/S1PR_1_ axis has been demonstrated to regulate the trafficking and migration of numerous types of immune cells, including T and B lymphocytes, NK cells, dendritic cells.^88^ Moreover, the S1P_1_ receptor signaling pathways have been reported to inhibit the pathological damage induced by the host innate and adaptive immune responses, thus attenuating the cytokine storm observed in influenza virus infection.^40^ In particular, Teijaro et al.^40^ demonstrated that, in mice infected with A/Wisconsin/WSLH34939/09 influenza virus, S1P_1_ receptor subtype regulates a crucial signaling loop fundamental for the initiation of cytokine storm in respiratory endothelial cells. The administration of S1P_1_ agonist blunted cytokine storm, by significantly inhibiting secretion of cytokines and chemokines associated with influenza virus-induced pathology, such as IFN-α, CCL2, IL-6, TNFα, and IFN-γ.^40^ Notably, in endothelial cells, suppression of early innate immune responses through S1P_1_ signaling has been found to decrease mortality during influenza virus infection in mice.^40^ Interestingly, in a later work by the same group, activation of S1P_1_ signaling has been demonstrated to block cytokine and chemokine production, as well as immune cell activation and recruitment in the lungs of mice infected with the H1N1 WSN strain of influenza virus.^90^ Moreover, S1P_1_ agonism has been found to reduce cytokine storm independently of TLR3 and TLR7 signaling, as well as of multiple endosome and cytosolic innate pathogen-sensing pathways.^90^ In contrast, S1P_1_R agonism has been found to suppress cytokine and chemokine production by targeting MyD88 (myeloid differentiation primary response gene 88)/TRIF (TIR-domain-containing adapter-inducing IFN-β) signaling, two common actors with NF-κB pathway.^90^ However, S1P_1_R agonism is likely to modulate other signaling pathways that have not yet identified.

Thus, based on the effects of S1P receptor signaling on multiple immunological processes indicating such pathway a promising for the modulation of harmful inflammatory responses, the application of therapies targeting S1P and S1P signaling may be repurposed for immune-mediated disorders and inflammatory conditions, such as COVID-19. For instance, SP1 agonists, approved for multiple sclerosis, such as fingolimod, might be used as therapeutic tools to dampen cytokine and chemokine responses in those patients displaying excessive immune responses. To date, only one non-randomized phase II clinical trial is underway to establish the efficacy of fingolimod in the treatment of COVID-19 (NCT04280588) (typing COVID-19 and fingolimod on clinicaltrials.gov).

## Concluding remarks: a glimpse into the future

The COVID-19 pandemic, induced by the novel SARS-CoV-2, represents one of the greatest global public health emergencies since the pandemic influenza outbreak of 1918 and provides an unprecedented challenge for the identification of both preventive and therapeutic drugs. In particular, vaccines and effective therapeutics to tackle this novel virus are urgently needed. Fortunately, in the last decade vaccine technology has significantly evolved, with the development of several RNA and DNA vaccine candidates, licensed vectored vaccines, recombinant proteins and cell culture-based vaccines for many indications.^91^ Moreover, given the similarities of SARS-CoV-2 with SARS-CoV, the ideal target for the vaccine, the spike S protein on the surface of the virus required for viral entry, has been quickly identified, providing a target antigen to incorporate into advanced vaccine platforms. Thus, antibodies specifically targeting the S protein can block the binding of SARS-CoV-2 to the host ACE2 receptor, thus neutralizing the virus. However, given the lesson learned from SARS and MERS, the development of the vaccine against SARS-CoV-2 is likely to be an uphill road with several obstacles. In fact, several vaccines for SARS-CoV, including recombinant S protein-based vaccines, have been already developed and tested in animal models, but many did not produce sterilizing immunity in animal models and/or induced severe side effects, such as lung and liver damage.^92,93^ To date, no human CoV vaccines have been approved so far. Moreover, to complicate this scenario, it has to be still unveiled whether infection with CoVs induces long-lived antibody response protecting against the risk of relapsing infections. Thus, scientific community has to overcome several issues for the development of an effective and safe SARS-CoV-2 vaccine. In this regard, Ling et al. recently detected SARS-CoV-2-specific humoral and cellular immunity in 8 COVID-19 patients, recently become virus-free and consequently discharged.^94^ In addition, the neutralizing antibody titers have been significantly correlated with the numbers of nucleocapsid protein-specific T cells.^94^ Such evidence indicates that both B and T cells cooperate to protect the host from viral infection. Notably, despite the small sample size, this study laid a theoretical foundation for the diagnosis of infectious diseases, the tracing of past infections, as well as the development of therapeutic antibody drugs and the design of an effective vaccine. Consistently, Long et al. reported acute antibody responses to SARS-CoV-2 in a cohort of 285 patients with COVID-19.^31^ In particular, 19 days after symptom onset, 100% of patients have been tested positive for antiviral IgG and seroconversion for IgG and IgM have been reported to occur simultaneously or sequentially.^31^ Thus, serological testing might be useful to identify suspected patients with negative RT-PCR results as well as asymptomatic infections.^31^

However, the speed at which SARS-CoV-2 is spreading has emphasized the urgent need to identify alternative therapeutic strategies in order to contain viral infection and to attenuate the excessive host immune response during the lag of vaccine availability, especially in a scenario where the virus may become endemic and recurrent seasonal epidemics may occur. In this regard, several antiviral drugs, such as remdesivir, lopinavir and ritonavir, are currently tested in several clinical trials, either alone or in combination, and compassionate use of these drugs has already been reported for SARS-CoV-2.^95,96^ However, antiviral drugs might select for mutational escape, thus rendering this therapeutic approach ineffective. Moreover, still unconfirmed reports indicate sufficient pre-clinical rationale and evidence regarding the use of chloroquine and hydroxychloroquine as prophylactic agent,^97^ with evidence of safety from long-time use in clinical practice for the treatment of malaria and autoimmune diseases.^98^ However, their use needs further evidence and clinical evaluation. Chloroquine and hydroxychloroquine are known to potentially cause heart rhythm problems, that may be exacerbated whether combined with other drugs with similar effects on the heart, and induce adverse liver, kidney and cerebral effects.^99^ Thus, as discussed in this review, treatments addressing the immunopathology of the infection, such as immunomodulatory drugs approved for different clinical indications, have become a major focus. Such approaches show the advantage to override viral mutational escape and to exert less-selective pressure on viral population. Although the prospective of counteracting cytokine storm is compelling, a major limitation relies on the limited understanding of the immune signaling pathways triggering such process. Hence, future dissection of immune signaling pathways triggered by SARS-CoV-2 will provide novel insight on the effects of the virus on human immune system and may reveal relevant biological players that can be targeted to blunt cytokine storm. Notably, since it is well established that innate immune responses trigger the activation of multiple and redundant signaling pathways, an effective therapy may require to acting, at the same time, on multiple signaling pathways. In this regard, cocktails of immunomodulatory drugs, such as monoclonal antibody targeting a specific cytokine (e.g. TNF- inhibitors, IL-1-inhibitors, IL-6 inhibitors), corticosteroids (e.g. prednisone, methylprednisolone and dexamethasone), and S1PR_1_ agonists (e.g. fingolimod), rather than a single drug, might be more effective in the management of COVID-19, by exerting either synergic or additive effects. In this regard, it would be of key importance to assess whether patients with immune-mediated disorders treated with immunomodulatory drugs, such as cytokine blockers, are more resistant to the excessive immune response observed in COVID-19 patients and more protected against SARS-CoV-2-driven pneumonia. However, to date, no evidence reporting either decreased or increased risk of SARS-CoV-driven pneumonia has been documented in this patients and further investigations are required to verify this hypothesis.

Furthermore, another aspect to better investigate concerns the possibility that the uncontrolled immune response to viral infection may cause detrimental systemic effects on several physiological systems, such as the nervous, endocrine, renal and cardiovascular systems. Accordingly, it is likely that the massive cytokine and chemokine release may critically impact on these physiological systems, thereby inducing both short- and long-term detrimental effects. As an example, the neuro-invasive potential of SARS-CoV and MERS-CoV has been previously reported.^100^ Thus, given the high similarity between SARS-CoV and SARS-CoV-2, it is likely that this latter displays a similar potential.^101–103^ As a matter of fact, a study carried out in 214 COVID-19 patients reported that about 88% of severe COVID-19 cases showed neurologic manifestations, such as acute cerebrovascular diseases and impaired consciousness.^104^

Finally, beside the putative long-term effects directly induced by SARS-CoV-2 infection, another key issue to address concerns the long-term effects of empirical and experimental treatments in COVID-19 patients. In this regard, a study carried out in 25 recovered SARS patients, recruited 12 years after the viral infection, reported significant differences in the serum metabolomes in recovered SARS patients compared with controls.^105^ In particular, a significant metabolic alteration — increased levels of phosphatidylinositol and lysophosphatidylinositol — has been found to coincide with the effect of methylprednisolone administration,^105^ thus suggesting that high-dose pulses of steroid treatment may induce long-term systemic damage associated with serum metabolic alterations.^105^

Therefore, all the challenges discussed above highlight some of the major gaps in our knowledge of COVID-19 clinical spectrum, underlying immune signaling pathways, systemic effects, and long-term pathological signatures, which need to be urgently fulfilled by future investigations.
